# A systematic review and meta-analysis of the nutrient content of preterm and term breast milk

**DOI:** 10.1186/1471-2431-14-216

**Published:** 2014-08-30

**Authors:** Dominica A Gidrewicz, Tanis R Fenton

**Affiliations:** 1 Department of Pediatrics, University of Calgary, 2888 Shaganappi Trail NW, Calgary, AB T3B 6A8, Canada; 2 Nutrition Services, Alberta Health Services, Department of Community Health Sciences, Alberta Children’s Hospital Research Institute, Faculty of Medicine, University of Calgary, 3rd Floor, 3280 Hospital Drive NW, Calgary, AB T2N 4Z6, Canada

**Keywords:** Human milk, Lactation, Breast milk, Infant, Premature

## Abstract

**Background:**

Breast milk nutrient content varies with prematurity and postnatal age. Our aims were to conduct a meta-analysis of preterm and term breast milk nutrient content (energy, protein, lactose, oligosaccharides, fat, calcium, and phosphorus); and to assess the influence of gestational and postnatal age. Additionally we assessed for differences by laboratory methods for: energy (measured vs. calculated estimates) and protein (true protein measurement vs. the total nitrogen estimates).

**Methods:**

Systematic review results were summarized graphically to illustrate the changes in composition over time for term and preterm milk. Since breast milk fat content varies within feeds and diurnally, to obtain accurate estimates we limited the meta-analyses for fat and energy to 24-hour breast milk collections.

**Results:**

Forty-one studies met the inclusion criteria: 26 (843 mothers) preterm studies and 30 (2299 mothers) term studies of breast milk composition. Preterm milk was higher in true protein than term milk, with differences up to 35% (0.7 g/dL) in colostrum, however, after postnatal day 3, most of the differences in true protein between preterm and term milk were within 0.2 g/dL, and the week 10–12 estimates suggested that term milk may be the same as preterm milk by that age. Colostrum was higher than mature milk for protein, and lower than mature milk for energy, fat and lactose for both preterm and term milk. Breast milk composition was relatively stable between 2 and 12 weeks. With milk maturation, there was a narrowing of the protein variance. Energy estimates differed whether measured or calculated, from −9 to 13%; true protein measurement vs. the total nitrogen estimates differed by 1 to 37%.

**Conclusions:**

Although breast milk is highly variable between individuals, postnatal age and gestational stage (preterm versus term) were found to be important predictors of breast milk content. Energy content of breast milk calculated from the macronutrients provides poor estimates of measured energy, and protein estimated from the nitrogen over-estimates the protein milk content. When breast milk energy, macronutrient and mineral content cannot be directly measured the average values from these meta-analyses may provide useful estimates of mother’s milk energy and nutrient content.

## Background

Breast milk composition is variable. While breast milk is the recommended feeding for all infants [1-3], including preterm infants [2,4,5], its variable composition makes estimating nutrient intakes difficult. Milk produced by mothers who deliver prematurely is well known to be higher in protein [4,5]. Milk composition changes with postnatal age; protein content decreases over weeks after birth [6]. Breast milk fat and energy content varies from the start to the end of a feeding, and follows a diurnal pattern in both term [7,8] and preterm milk [8,9].

In addition, there are several reasons for the variability in the values of breast milk composition due to laboratory methods used for the analysis. Two approaches have been used to quantify energy in breast milk: a) direct energy quantification by combusting in a bomb calorimetry and b) calculated energy estimates using Atwater energy multiplication factors for the macronutrients: protein, fat, and carbohydrate [10]. Two methods used to estimate protein content include a) direct quantification of the true protein content and b) quantification of the nitrogen (assuming that all nitrogen is protein, rather than recognition that some is in non-protein nitrogen compounds [11-13].

Thus we conducted a systematic review and meta-analysis of observational studies on the composition of breast milk nutrient content (energy, macronutrient (protein, lactose, fat)) and mineral content (calcium, phosphorus). We hypothesized that the composition of breast milk depends on four variables, which include: gestational stage (premature birth), postnatal age, calculated versus measured energy estimates, and protein method (true protein versus total nitrogen). We conducted the meta-analyses of breast milk composition stratified by these 4 factors (gestational stage; postnatal age; energy estimation method [measurement vs. calculation]; and protein estimation method [true protein versus total nitrogen]), to determine whether any or all of these factors should be considered when estimating breast milk nutrient content.

## Methods

### Literature search

In an attempt to find all published literature on the topic, studies relating to breast milk content in premature and mature milk were identified through computerized searches. First searches were conducted in MedLine and Embase for studies published in any language using the following Medical Subject Headings and text words: human, milk, lactation, breast milk, breast milk, protein, energy calories, lactose, oligosaccharide(s), fat, calcium, phosphorus, and infant, premature, preterm, neonate, or newborn, independently by the two investigators (DG and TRF) in March 2014. In an effort to include all available studies, a Web of Science search was conducted for all papers that cited the references Schanler et al. 1980 [14] and Atkinson SA et al. 1980 [15] (by DG). A grey literature search was also conducted to avoid reporting bias and look for unpublished literature (by DG) in March 2014. We reviewed the reference lists of included papers.

The inclusion criteria were: studies that reported on analysis of energy, macronutrient (protein, fat, lactose) and/or mineral (calcium, phosphorus) content in the breast milk of healthy, term (37–42 wk of gestation) and preterm (<37 wk of gestation) infants, if the data were reported categorized by postnatal age and term versus preterm status. Review articles and commentaries were excluded. Studies conducted in developing countries (i.e. outside North America, Europe, Australia, Israel and Japan [16]) were excluded in an attempt to exclude mothers with suboptimal nutritional status. The Meta-analysis Of Observational Studies in Epidemiology (MOOSE) Proposal for Reporting [17] was used to guide this study.

### Data extraction

All article titles were examined for potential fit to the inclusion criteria by the two reviewers (DG and TRF). When the title was not clear regarding the potential fit, then the abstract was reviewed; when the abstract was not clear whether the study fit the inclusion criteria, the paper was reviewed. In studies where the data was presented in a non-numerical format, and thus not possible to include in a meta-analysis, efforts were made (by DG) to contact the author to obtain these data. If no response was received to the request or the author was unable to provide additional data, the study was not included in the meta-analysis. Data were extracted by DG and checked for accuracy by TRF.

Since breast milk fat content varies between fore and hind milk [6,7] and diurnally between early and later in the day [7-9], to obtain accurate estimates we limited the meta-analyses for energy and fat to 24-hour breast milk collections. This requirement was not placed on the other analyses since the differences between fore and hind milk and diurnally in protein are not of an important magnitude [6,7].

### Analysis

Meta-analyses were carried out on studies that reported the following outcomes in either healthy, term or preterm delivering mothers: total energy (kcal/dL), protein (g/dL), fat (g/dL), lactose content (kcal/dL), calcium (mg/dL), and phosphate (mg/dL). Data was grouped into the following time points: 1–3 days (representing colostrum), 4 to 7 days, week 2 (day 8–14), week 3–4 (days 15–28), week 5–6 (days 29–42), week 7–9 (days 43 – 63), week 10–12 (days 64 – 84). We continued the meta-analyses to 12 weeks since age-specific data was sparse for the analyses after this age.

To examine whether the two energy measures, bomb calorimetry and calculation methods, estimated different energy contents, separate meta-analyses were prepared for each energy estimation method and compared. Energy reported as kilojoules was converted to kilocalories by dividing by 4.184.

Historically, protein in breast milk has been estimated in two different ways: including or excluding the non-protein nitrogen. Thus, we conducted two meta-analyses of protein for the available data: an estimate of protein based on the assumption that all of the nitrogen is protein and a true protein estimate which excludes the non-protein nitrogen. When only total protein was only reported in terms of total nitrogen, total protein was calculated by multiplying the nitrogen by 6.25 [12-14,18-23].

Mineral data reported as millimoles was converted to milligrams by multiplying by the molecular weight. Breast milk data reported per kilogram was converted to per liter by dividing by 1.032 [24].

The nutrient content meta-analyses were calculated as weighted averages and pooled standard deviation for each time period, for preterm and term breast milk. For statistical comparisons, t-tests were used to compare preterm and term milk composition. Given the multiple comparisons made in this study, an approximate Bonferroni adjustment was made, and the p-value for statistical significance used was 0.001.

## Results

**Figure 1:**
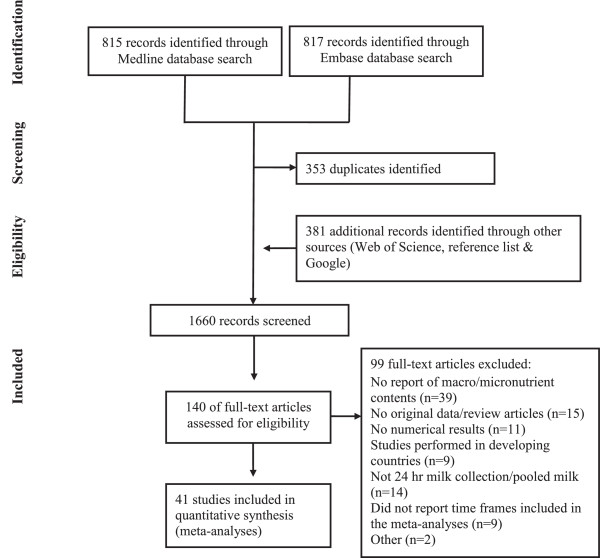
Flow diagram of the literature search process.

A total of 41 studies were included in the analysis: 26 (843 mothers) and 30 (2299 mothers) studies reporting on preterm and term breast milk composition, respectively (Table 1). Attempts were made to contact authors of nine studies, we received replies from four, but no additional information was received for the meta-analyses. Ninety-nine studies were excluded for reasons provided in Figure 1: no original data/review articles [25-39], studies performed in developing countries [40-48], no numerical results [49-59], not 24-hour milk collection/pooled milk (required only for energy and fat contents) [7-9,60-70], no report of macro/micronutrient contents [36,71-107], did not report time frames used in the meta-analyses [108-116], other [117,118]. Energy was estimated in 11 studies using bomb calorimetry [11,12,18,119-126] and in five studies by calculation using values for the energy contributions from fat, protein, and carbohydrate [6,19,22,121,127]. Protein was estimated based on total nitrogen in 23 studies [6,11-14,18-23,120,122,123,125,128-135] and as a true protein estimate in 15 studies [11-14,18,19,121-123,127-129,136-139]. A summary of the meta-analyses breast milk composition at various postnatal ages for energy, protein, fat, calcium and phosphorus is outlined in Table 2.

**Table 1:** Studies included in the meta-analysis

**Reference**	**Site**	**Subjects**				**Reported outcomes**
		** Preterm**	** n**	** Term**	** n**	
Anderson et al., 1983 [18]	US	28-36 weeks	14	37-42 weeks	9	E, Pro, fat
Arnold et al., 1987 [130]	Australia	26-33 weeks	6	38-40 weeks	7	Pro, lactose
Atkinson et al., 1980 [15]	Canada	26-33 weeks	13	38-40 weeks	10	Ca, P
Atkinson et al., 1981 [19]	Canada	BW < 1300 g	7	-		E, Pro, lactose, fat
Bejiers et al., 1992 [128]	Netherlands	25.7–30.9 weeks	30	-		Pro
Britton et al., 1986 [137]	US	25-35 weeks	70	38-42 weeks	38	Pro
Butte et al., 1984 [13]	US	< 37 weeks	8	≥ 37 weeks	13	Pro, Ca, P
Butte et al., 1984 [122]	US	-		Term	40	E, Pro, fat
Butte NF et al., 1990 [123]	US	-		39.9 ± 0.9 weeks	40	E, Pro, lactose, fat
Coppa et al., 1993 [140]	Italy	-		Term	46	Lactose, oligo
Coppa et al., 1997 [141]	Italy	27-35 weeks	26	-		Oligo
Corvaglia et al., 2008 [20]	Italy	26-32 weeks	55	37-41 weeks	69	Pro
Cregan MD, 2002 [135]	Australia	31-35 weeks	22	> 38 weeks	16	Pro, lactose
Ehrenkranz et al., 1984 [142]	US	26-33 weeks	21	-		fat
Faerk et al., 2001 [133]	Denmark	< 32 weeks	101	-		Pro
Ferris et al., 1988 [21]	US	-		> 37 weeks	12	Pro, lactose
Garza et al., [124]	US	-		Term	10	E
Gabrielli et al., 2011 [143]	Italy	25-30 weeks	63	-		Lactose, oligo
Gross et al., 1980 [131]	US	28-36 weeks	33	38-42 weeks	18	Pro, lactose, Ca
Guerrini et al., 1981 [144]	Italy	29-37 weeks	25	38-42 weeks	47	fat
Hibberd et al., 1982 [11]	UK, Germany	-		> 37 weeks	10	E, Pro, lactose, fat, Ca
Hosoi et al., 2005 [134]	Japan	-		Term	114	Pro
Hurgoiu et al., 1986 [145]	Romania	27-34 weeks	28	-		Ca
Itabashi et al., 1999 [129]	Japan	26-33 weeks	15	-		Pro, lactose, Ca, P
Lepage et al., 1984 [120]	Canada, US	26-36 weeks	32	> 37 weeks	19	E, Pro
Lemons et al., 1982 [12]	US	27-37 weeks	20	39-41 weeks	7	E, Pro, lactose, fat, Ca, P
Maas et al., 1998 [22]	Netherlands	25-29 weeks	79	-		E, Pro, lactose, fat
Michaelsen et al., 1994 [139]	Denmark	-		37-41weeks	91	Pro, fat, lactose
Montagne et al., 1999 [136]	France	< 37 weeks	46	> 37 weeks	28	Pro
Motil et al., 1997 [125]	US	-		38-42 weeks	10	E, Pro
Nommsen et al., 1991 [127]	US	-		Term	58	E, Pro, fat
Reinken et al., 1985 [132]	Germany	28-33 weeks	16	38-40 weeks	24	Pro
Saarela et al., 2005 [6]	Finland	31.4 ± 3 weeks	36	40.2 ± 1.4 weeks	53	E, Pro, lactose, fat
Sadurskis et al., 1998 [119]	Sweden			Term	23	E
Sanchez-Pozo et al., 1986 [138]	Spain			Term	209	Protein
Sann et al., 1981 [146]	France	26-35 weeks	41	38-41 weeks	61	Pro, lactose, fat, Ca, P,
Schanler et al., 1980 [14]	US	29.7 ± 0.5 weeks	16	-		Pro, Ca
Thomas et al., 1986 [121]	US	30-34 weeks	20	-		E, Pro, lactose, fat
Yamawaki et al., 2005 [23]	Japan	-		BW > 2500 g	1180	Pro, lactose, Ca, P
Viverge et al., 1990 [147]	France	-		Term	15	Lactose, oligo
Wood et al., 1988 [126]	US	-		37-42 weeks	22	E
Total			843		2299	

E = energy, Pro = protein, oligo = oligosaccharides, Ca = calcium, P = phosphate, BW = birth weight, g = gram, UK = United Kingdom, US = United States.

**Table 2:** Meta-analysis summary estimates of breast milk composition per 100 milliliters at various postnatal ages (mean (+/−2 standard deviations))

**Preterm**	**Energy (kcal)**	**Protein (g)**	**Fat (g)**	**Calcium (mg)**	**Phosphorus (mg)**
1^st^ week	60 (45–75)	2.2 (0.3-4.1)	2.6 (0.5-4.7)	26 (9–43)	11 (1–22)
2^nd^ week	71 (49–94)	1.5 (0.8-2.3)	3.5 (1.2-5.7)	25 (11–39)	15 (8–21)
Week 3/4	77 (61–92)	1.4 (0.6-2.2)	3.5 (1.6-5.5)	25 (13–36)	14 (8–20)
Week 10/12	66 (39–94)	1.0 (0.6-1.4)	3.7 (0.8-6.5)	29 (19–38)	12 (8–15)
**Term**	**Energy (kcal)**	**Protein (g)**	**Fat (g)**	**Calcium (mg)**	**Phosphorus (mg)**
1^st^ week	60 (44–77)	1.8 (0.4-3.2)	2.2 (0.7-3.7)	26 (16–36)	12 (6–18)
2^nd^ week	67 (47–86)	1.3 (0.8-1.8)	3.0 (1.2-4.8)	28 (14–42)	17 (8–27)
Week 3/4	66 (48–85)	1.2 (0.8-1.6)	3.3 (1.6-5.1)	27 (18–36)	16 (10–22)
Week 10/12	68 (50–86)	0.9 (0.6-1.2)	3.4 (1.6-5.2)	26 (14–38)	16 (9–22)

Estimates as +/− 2 standard deviations assumed no skew. Energy values were bomb calorimeter measured values except for 10–12 weeks, which were calculated values. Protein values are true measured protein, not based on total nitrogen content.

### Energy measurement vs. calculation from the macronutrients

**Figure 2:**
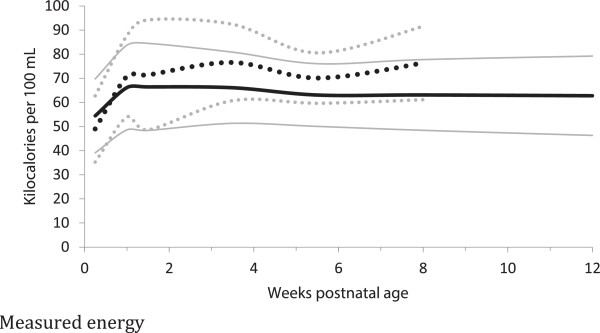
**Measured Energy distribution of preterm and term breast milk by postnatal age over the first 12 weeks of lactation, weighted mean and 95% reference interval.** Preterm milk ^**….**^ Term milk **----** : mean +/- 2 standard deviations.

**Figure 3:**
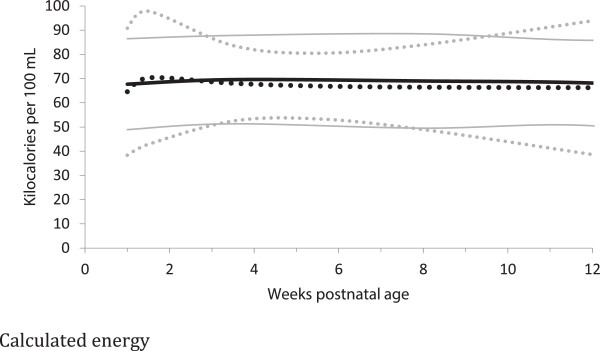
**Calculated Energy estimates distribution of preterm and term breast milk by postnatal age over the first 12 weeks of lactation, weighted mean and 95% reference interval.** Preterm milk ^….^ Term milk ---- : mean +/- 2 standard deviations.

In the comparison between measured and calculated energy contents of milk, measured estimates were −6 to 10 kcal/dL (−9 to 13%) greater than the calculated analyses (Table 3, Figures 2 and 3), but only four differences (preterm milk at weeks 3–4 and 7–9, term milk at weeks 7–9 and 10–12 weeks) met the adjusted statistical significance criteria (i.e. p < 0.001). Most of the preterm measured energy estimates had less than 30 subjects (Table 3), and while the calculated energy estimates generally had higher numbers; none of the studies that reported calculated energy estimates had any data for the first few postnatal days (Figure 2 and 3, Table 3).

**Table 3:** Meta-analysis results of preterm and term breast milk energy content over time from measured and calculated estimates

**Comparison: Bomb calorimetry energy measurement (kcal/dL)♦**								
	**Preterm**			**Term**			**Preterm & term compared**	
**Time frame:**	**mean**	**SD**	**n**	**Mean**	**SD**	**n**	**% difference**	**p-value**
d 1-3	49	7	12	54	8	19	−10	0.34
d 4-7	71	9	52	66	9	37	7	0.02
week 2	71	12	53	66	9	34	7	0.04
week 3-4	77	8	27	66	8	97	16	< 0.00001*
week 5-6	70	5	14	63	7	40	11	< 0.00001*
week 7-9	76	8	11	63	7	77	21	< 0.00001*
week 10-12	-	-	-	63	8	83	-	-
Energy meta-analysis was limited to 24 hour collections								
**♦** References: [11,12,18,119-126]								
**Comparison: Calculated energy content (kcal/dL)♦♦**								
	**Preterm**			**Term**			**Preterm & term compared**	
**Time frame:**	**mean**	**SD**	**n**	**Mean**	**SD**	**n**	**% difference**	**p-value**
d 1-3	-	-	-	-	-	-		
d 4-7	65	13	41	68	9.6	48	−5	0.21
week 2	70	14	95	-	-	-		
week 3-4	68	8.0	135	70	9.3	46	−2	0.26
week 5-6	67	6.9	79	-	-	-		
week 7-9	66	8.9	63	69	9.9	43	−4	0.16
week 10-12	66	14	14	68	9.0	95	−3	0.50
**♦♦** References: [6,19,22,121,127]								
**Comparison: Measured vs. calculated energy**								
	**Preterm**		**Term**					
	**Difference**	**% difference**	**p-value**	**Difference**	**% difference**	**p-value**		
d 1-3		-	-		-	-		
d 4-7	6	−9%	0.009	−2	2%	0.350		
week 2	1	−2%	0.66					
week 3-4	9	−11%	< 0.00001*	−3	5%	0.007		
week 5-6	3	−5%	0.11					
week 7-9	10	−13%	0.0009*	−6	9%	0.0003*		
week 10-12				−5	9%	0.0002*		

*Statistically significant difference. In compensation for multiple comparisons, an approximate Bonferroni adjustment was made and the p-value for statistical significance was < 0.001.

### Protein estimation method [true protein versus total nitrogen estimate]

**Figure 4:**
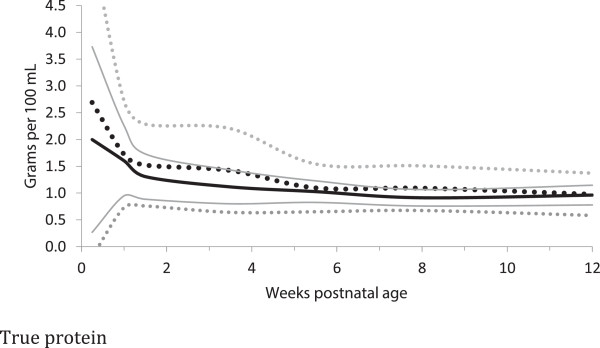
**True Protein content distribution of preterm and term breast milk in by postnatal age over the first 12 weeks of lactation, weighted mean and 95% reference interval.** Preterm milk ^**….**^ Term milk **----** : mean +/- 2 standard deviations.

**Figure 5:**
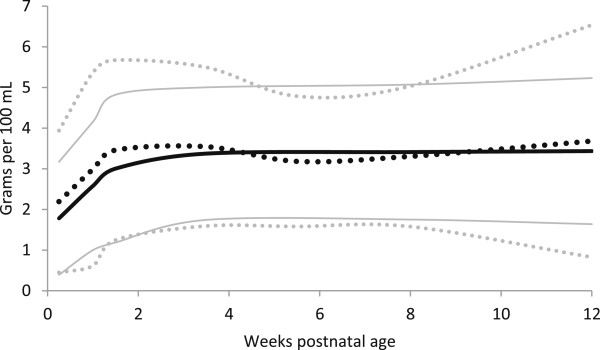
**Fat content distribution of preterm and term breast milk by postnatal age over the first 12 weeks of lactation, weighted mean and 95% reference interval.** Preterm milk ^**….**^ Term milk **----** : mean +/- 2 standard deviations.

Almost all of the differences in protein content, between the estimates of protein based on total nitrogen content and the measured true protein estimates were statistically significantly lower for the true protein measures for most time periods, for both term and preterm milk, (Table 4, Figures 4 and 5). The most common differences in quantity between the total nitrogen and true protein estimates was 0.3 g/dL (Table 4).

**Table 4:** Meta-analysis results of preterm and term breast milk protein content over time

**Comparison: True protein comparisons: Preterm vs. term (g/dL)♦**								
	**Preterm**			**Term**		**Preterm & term compared**		
**Time frame:**	**mean**	**SD**	**n**	**mean**	**SD**	**n**	**% difference**	**p-values**
d 1-3	2.7	1.5	141	2.0	0.9	108	35	< 0.00001*
d 4-7	1.7	0.5	165	1.6	0.3	185	7	0.005
week 2	1.5	0.4	191	1.3	0.2	256	16	< 0.00001*
week 3-4	1.4	0.4	92	1.1	0.2	194	27	< 0.00001*
week 5-6	1.1	0.2	38	1.0	0.1	85	7	0.0003
week 7-9	1.1	0.2	30	0.9	0.1	113	20	< 0.00001*
week 10-12	1.0	0.2	25	1.0	0.1	221	2	0.37
**♦** References: [11-14,18,19,121-123,127-129,136-139]								
**Comparison: Total protein comparisons: Preterm vs. term (g/dL)♦♦**								
	**Preterm**			**Term**		**Preterm & term compared**		
**Time frame:**	**mean**	**SD**	**n**	**mean**	**SD**	**n**	**% difference**	**p-values**
d 1-3	2.8	1.1	94	2.0	0.6	168	37	< 0.00001*
d 4-7	2.1	0.5	244	2.0	0.5	229	4	0.04
week 2	1.9	0.4	253	1.8	0.4	192	8	< 0.00001*
week 3-4	1.6	0.4	439	1.5	0.3	210	9	0.01
week 5-6	1.4	0.3	268	1.1	0.2	357	18	< 0.00001*
week 7-9	1.1	0.2	183	1.3	0.2	453	−10	< 0.00001*
week 10-12	1.3	0.3	18	1.2	0.2	109	12	0.07
**♦♦** References: [6,11-14,18-23,120,122,123,125,128-135]								
**Comparisons: True vs. Total protein ♦♦♦**								
	**Difference**	**% difference**	**p-value**	**Difference**	**% difference**	**p-value**		
d 1-3	0.1	4%	0.60	0	1%	0.91		
d 4-7	0.3	20%	< 0.00001*	0.4	24%	< 0.00001*		
week 2	0.4	26%	< 0.00001*	0.5	36%	< 0.00001*		
week 3-4	0.2	12%	< 0.00001*	0.4	31%	< 0.00001*		
week 5-6	0.3	27%	< 0.00001*	0.1	11%	< 0.00001*		
week 7-9	0	3%	0.35	0.3	37%	< 0.00001*		
week 10-12	0.3	32%	0.0002	0.2	20%	< 0.00001*		

**♦♦♦**Estimates based on true protein content versus the assumption that all nitrogen is protein.

*Statistically significant difference. In compensation for multiple comparisons, an approximate Bonferroni adjustment was made and the p-value for statistical significance was < 0.001.

### Gestational stage effect: preterm milk compared to term milk

In a comparison of the term versus preterm milk, most of the analytes (with the exception of fat and calculated energy) had some differences between the preterm and term milk composition that were statistically significant (Tables 3, 4, 5, 6, 7).

**Table 5:** Meta-analysis results of preterm and term breast milk fat, lactose and oligosaccharide content over time

	**Preterm**			**Term**			**Preterm & term compared**	
**Fat (g/dL)♦**								
**Time frame:**	**mean**	**SD**	**n**	**mean**	**SD**	**n**	**% difference**	**p-value**
d 1-3	2.2	0.9	76	1.8	0.7	74	23	0.002
d 4-7	3.0	1.2	111	2.6	0.8	136	16	0.002
week 2	3.5	1.1	158	3.0	0.9	48	15	0.01
week 3-4	3.5	1.0	180	3.4	0.8	127	5	0.12
week 5-6	3.2	0.8	95	3.6	1.1	20	−11	0.07
week 7-9	3.3	0.9	120	3.4	0.8	83	−3	0.38
week 10-12	3.7	1.5	22	3.4	0.9	95	7	0.31
Fat meta-analysis was limited to 24 hour collections.								
**♦** References: [6,11,12,18,19,22,121-123,125,127,142,144,146]								
**Lactose (kcal/dL)♦♦**								
**Time frame:**	**mean**	**SD**	**n**	**mean**	**SD**	**n**	**% difference**	**p-value**
d 1-3	5.1	0.7	95	5.6	0.6	59	−9	< 0.00001*
d 4-7	6.3	1.1	114	6.0	1.0	281	4	0.009
week 2	5.7	0.8	231	6.2	0.6	100	−8	< 0.00001*
week 3-4	6.0	0.5	225	6.7	0.7	193	−10	< 0.00001*
week 5-6	5.8	0.6	104	6.1	1.0	22	−6	0.06
week 7-9	6.3	0.4	123	6.5	0.5	646	−2	< 0.00001*
week 10-12	6.8	0.3	28	6.7	0.7	58	2	0.47
**♦♦**References: [6,11,12,19,21-23,121,123,129-131,135,140,143,146,147]								
**Oligosaccharides (g/dL) ♦♦♦**								
**Time frame:**	**mean**	**SD**	**n**	**mean**	**SD**	**n**	**% difference**	**p-value**
d 1-3	-	-	-	1.6	0.2	9	-	-
days 4-7	2.1	0.4	89	1.9	0.4	93	12	0.0009
week 2 (days 7–14)	2.1	0.5	89	1.9	0.4	54	7	0.004
week 3–4 (days 15–30)	1.7	0.3	152	1.6	0.3	46	12	0.27
week 5-6	-	-	-	1.4	0.3	46	-	-
week 7-9	-	-	-	1.3	0.3	46	-	-
week 10-12	-	-	-	-	-	-	-	-
**♦♦♦**References: [140,141,143,147].								

*Statistically significant difference. In compensation for multiple comparisons, an approximate Bonferroni adjustment was made and the p-value for statistical significance was < 0.001.

**Table 6:** Meta-analysis results of preterm and term breast milk mineral content over time

	**Preterm**			**Term**			**Preterm & term compared**	
**Calcium (mg/dL)♦**								
**Time frame:**	**mean**	**SD**	**n**	**mean**	**SD**	**n**	**% difference**	**p-value**
d 1-3	25	9	50	26	6	26	−3	0.6
d 4-7	27	9	88	26	4	86	5	0.34
week 2	25	7	116	28	7	100	−10	0.002
week 3-4	25	6	108	27	5	85	−8	0.01
week 5-6	28	6	41	25	6	223	11	0.004
week 7-9	30	6	37	26	6	363	15	0.0002*
week 10-12	29	5	30	27	3	13	6	0.17
**♦** References: [11-15,23,88,129,131,145,146]								
**Phosphate (mg/dL)♦♦**								
**Time frame:**	**mean**	**SD**	**n**	**mean**	**SD**	**n**	**% difference**	**p-value**
d 1-3	10	7	7	11	3	6	−14	0.62
d 4-7	13	4	79	13	4	86	3	0.50
week 2	15	3	67	15	4	90	−4	0.44
week 3-4	14	3	56	16	3	75	−14	0.0004*
week 5-6	13	2	33	16	3	213	−16	< 0.0001*
week 7-9	14	2	29	16	3	363	−13	0.002
week 10-12	12	2	22	14	3	13	−19	0.03
**♦♦** References: [12,13,15,23,88,129,131,146]								

*Statistically significant difference. In compensation for multiple comparisons, an approximate Bonferroni adjustment was made and the p-value for statistical significance was < 0.001.

**Table 7:** The milk maturity effect: Comparison of colostrum versus mature milk

	**Energy (measured)**		**Protein (true protein)**		**Fat**		**Lactose**	
	**Preterm**	**Term**	**Preterm**	**Term**	**Preterm**	**Term**	**Preterm**	**Term**
Colostrum	49	54	2.7	2.0	2.2	1.8	5.1	5.6
Mature milk	73	63	1.1	1.0	3.3	3.4	6.2	6.5
Difference	49%	16%	−61%	−52%	50%	93%	21%	16%
p-value	<0.00001*	<0.00001*	<0.00001*	<0.00001*	<0.00001*	<0.00001*	<0.00001*	<0.00001*
	**Calcium**		**Phosphate**					
	**Preterm**	**Term**	**Preterm**	**Term**				
Colostrum	25	26	9.5	11				
Mature milk	29	26	12.8	16				
Difference	13%	−2%	35%	41%				
p-value	0.003	0.62	0.002	0.001				

*met our approximate Bonferroni adjusted p-value criteria for statistical significance was < 0.001.

Colostrum was milk collected in the first 3 days, mature milk was collected between 5 to 12 weeks. The difference values less than 100% reflect lower values for mature milk, differences greater than 100% reflect higher values for colostrum compared to mature milk.

The energy content of preterm milk was similar to term milk at all postnatal ages, with three significant differences for the bomb calorimetric methods between 3 to 9 weeks; with differences of −10-21% (Table 3, Figures 2 and 3). We found no measured energy content data on preterm milk after 9 weeks.

Preterm milk was higher in true protein than term milk, with maximum mean differences up to 35% (0.7 g/dl) in the first few days after birth (Table 4, Figure 4). However, after postnatal day 3, most of the differences in true protein between preterm and term milk were within 0.2 g/dL or less, and the week 10–12 estimates suggested that term milk may be the same as preterm milk by that age. The estimates of protein based on total nitrogen suggested differences between preterm and term milk as high as 37% (0.8 g/dl) in the first few days, however after day 3, the most common difference between preterm and term protein estimates based on total nitrogen was 0.1 g/dL (Table 4).

The fat content of the preterm milk did not differ statistically (all p-values > 0.001) between preterm and term milk at any point in time, even though preterm milk was 23% higher than term milk (non-significant) in the first few days of life (Table 5, Figure 5).

**Figure 6:**
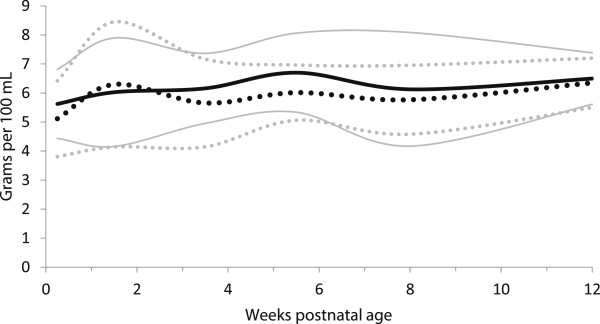
**Lactose content distribution of preterm and term breast milk by postnatal age over the first 12 weeks of lactation, weighted mean and 95% reference interval.** Preterm milk ^**….**^ Term milk **----** : mean +/- 2 standard deviations.

**Figure 7:**
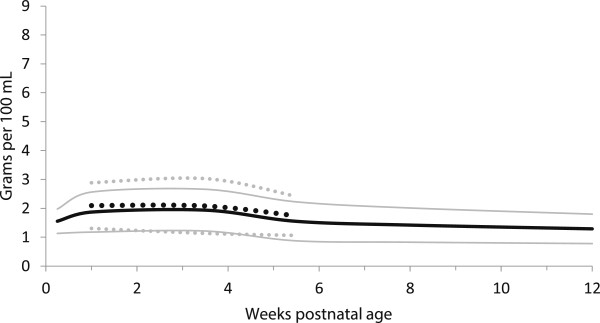
**Oligosaccharide content distribution of preterm and term breast milk oligosaccharide content in by postnatal age over the first 12 weeks of lactation, weighted mean and 95% reference interval.** Preterm milk ^**….**^ Term milk **----** : mean +/- 2 standard deviations.

Lactose was significantly lower in preterm milk compared to term milk, in the first 3 days and at a few later time points (Table 5, Figure 6). The general pattern of oligosaccharides showed similarities between preterm and term milk, although there was limited data for preterm milk (data only on days 4 – week 4) (Table 5, Figure 7). One difference was statistically significant for days 4–7 when preterm milk was 12% higher than term milk.

**Figure 8:**
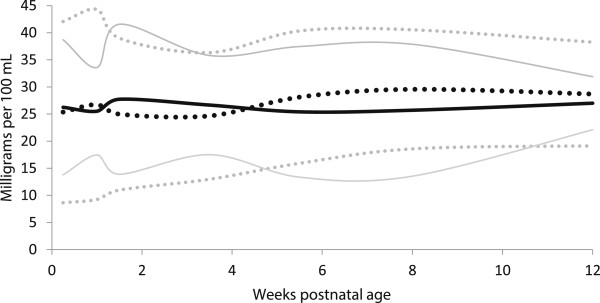
**Calcium content distribution of preterm and term breast milk calcium content in by postnatal age over the first 12 weeks of lactation, weighted mean and 95% reference interval.** Preterm milk ^**….**^ Term milk **----** : mean +/- 2 standard deviations.

**Figure 9:**
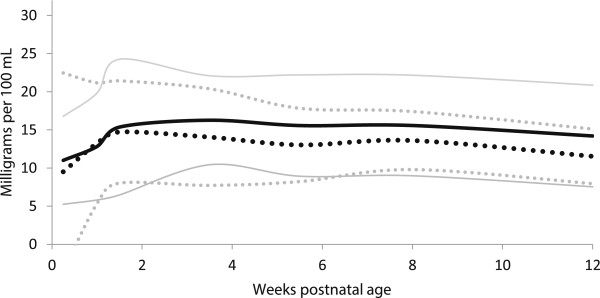
**Phosphate content distribution of preterm and term breast milk by postnatal age over the first 12 weeks of lactation, weighted mean and 95% reference interval.** Preterm milk ^**….**^ Term milk **----** : mean +/- 2 standard deviations.

The minerals, calcium and phosphate, were mostly similar between preterm and term milk. (Table 6, Figures 8 and 9).

### The milk maturity effect

In general, the meta-analyses of breast milk composition revealed relatively stable milk content between 2 and 12 weeks, after the initial fluctuations as the milk changed from colostrum to more mature milk (Tables 3, 4, 5, 6 and 7, Figures 2, 3, 4, 5, 6, 7, 8 and 9). The composition of colostrum compared to more mature milk (5 to 12 weeks) differed for all of the macronutrients by 16% or more (Table 2, Figures 2, 3, 4, 5, 6 and 7). Compared to colostrum, mature milk protein content decreased dramatically while fat increased by approximately one half in preterm milk or doubled in term milk. Measured energy and lactose were higher in mature milk compared to colostrum (Tables 3 and 5 Figures 2 and 6).

With milk maturation, there was a notable narrowing of the true protein variance in preterm milk, from the wide estimated 0 to 5.7 g/dL reference interval (+/− 2 standard deviations) in colostrum to the narrower mature milk estimated 0.6 to 1.4 g/dL at 12 weeks.

## Discussion

Much has been written about the differences between preterm and term breast milk, particularly about the nutritional superiority of preterm milk. This meta-analysis revealed more similarities than differences between preterm and term milk for energy, fat, oligosaccharides, calcium, and phosphorus. Gestational age (preterm vs term milk); postnatal age; protein estimation method [true protein versus total nitrogen estimate] and energy estimation method [measured versus calculated] were each found to identify important differences in breast milk content. Thus these factors should be considered when estimating breast milk nutrient content and in designing future studies to analyze breast milk nutrient content.

For energy, the differences between measured and calculated estimates of breast milk composition were only significantly different at three time points for preterm milk, however, the differences were as high as 10 kcal/dL (13%), which are likely clinically important differences. This data suggests that measured energy content of breast milk is superior to calculated methods.

It is possible that errors in the calculation of energy content of milk could be due to the various conversion factors used to calculate the energy contributions of the macronutrients and also from assuming that all of the nitrogen was protein [6,19,22] and that the only carbohydrate was lactose [6,19,121], which would contribute to an over- and an under-estimation, respectively, of the energy content of the milk [6].

The mean protein in early preterm milk was higher than in term milk at some time points during the first weeks, but also of importance, the variability of the protein content in preterm milk was twice that of term milk at most time points. The decrease in protein content and variance with postnatal age for preterm and term milk were similar over time. Although the differences in protein content between preterm and term milk were statistically significant for several time points, the differences may be only likely of clinical importance in the first few postnatal days. The meta-analysis revealed that protein content of preterm early milk may be very low in some mothers, based on the calculated reference intervals (mean +/− two (1.96) standard deviations, assuming that the milk composition was not skewed) of 0 to 5.6 g/dl. However biological parameters often are skewed. Further research is needed to describe the preterm milk protein distribution, range, and distribution symmetry.

The most dramatic changes from colostrum to mature milk was the decrease in protein and increase in fat, in both preterm and term milk, as well as the increase in energy in preterm milk (Table 7). There is evidence that the protein content of breast milk continues to decrease over time after birth, as revealed by analyses of donor breast milk reports that donated breast milk contains on average 0.9 grams of protein per 100 mL [110,113,148]. One of these studies of donated milk assessed the milk protein content at 8 months of postnatal age, and found the protein was 0.7 g/dL [110]. These studies did not meet our inclusion criteria since the milk from both preterm and term delivering mothers were combined [110,113,148].

Some researchers presented their estimates of breast milk protein content based on the total nitrogen, assuming that all of the nitrogen represented protein [6,11-14,18-23,120,122,123,125,128-135], some presented both protein estimates [11-14,18,128,129], while other researchers reported only true protein estimates [11-14,18,19,121-123,127-129,136-139]. The protein meta-analysis estimates based on total protein were almost uniformly higher than the true protein estimates, which suggest that these two approaches should not be averaged together. It has been suggested that the higher protein content of colostrum and early milk compared with later postnatal ages may not be digestible since much of this early non-protein nitrogen is non-digestible lactoferrin and IgA [128,149]. If some of this “protein” is not digestible, then it would not be available to meet nutritional protein needs.

This study was limited by the availability of results from the individual studies, and the various milk collection and analysis methods used. The minor undulations in the graphs may not represent real changes in breast milk nutrient content, but be due to differences between the studies and their methods. Another limitation was the limited sample sizes for some of the analyses.

## Conclusion

The protein content of breast milk decreases after birth to be less than half of the colostrum content by 6 weeks. Most of the differences in true protein between preterm and term milk were within 0.2 g/dL, and by 3 months of age, term milk may have the same protein content as preterm milk. The four parameters assessed in this study (postnatal age, gestational stage (preterm versus term), protein estimated from nitrogen versus measured protein content, and energy calculated from macronutrients versus measured using bomb calorimetry) were all found to be important predictors of breast milk content.

This meta-analysis evidence revealed that breast milk is highly variable between individuals. If breast milk energy macronutrient and mineral content cannot be directly analyzed for the individual mother and infant, the average values from these meta-analyses may provide useful estimates of the milk content.

For future research, our meta-analyses suggest that breast milk energy content calculated from the macronutrients provide poor estimates of measured energy and that protein estimated from the nitrogen over-estimates the true protein milk content.

## Competing interests

The authors declare that they have no competing interests.

## Authors’ contributions

DG and TRF independently searched the literature, DG attempted to contact authors when the data was not included in a form that could be extracted from the papers, DG extracted the data; TRF checked the data for accuracy and performed the meta-analyses. DG wrote the first draft of the paper and both authors contributed to the analysis and writing of the manuscript. Neither author has any conflicts of interest. All authors read and approved the final manuscript.

## Pre-publication history

The pre-publication history for this paper can be accessed here:

http://www.biomedcentral.com/1471-2431/14/216/prepub
